# Progressive Calcification

**Published:** 1896-05

**Authors:** Junius E. Cravens


### Progressive Calcification.

BY JUNIUS E. CRAVENS, D.D.S.

Read before the Mississippi Valley Dental Society, April 16, 1896.

A pulp that can live without protection should be protected. Pulps that can not live without protection were better destroyed. This law was promulgated on the Mount of Olives nearly two thousand years ago, by one who said "Unto every one that hath shall be given, and he shall have abundance. But from him that hath not shall be taken away even that which he hath." It is the oldest of the laws—the survival of the fittest.

The irrepressible activity of the dental pulp as a builder should not be overlooked nor undervalued. The philosophy of Nature does not require that completed dentine shall be nourished any more than that enamel shall be similarly favored. When in the process of development dentine has attained a maximum thickness or extent for practical purposes, regular activity of the odontoblasts is at an end; all subsequent activity of these elements is secondary and spasmodic, regional, responsive only to special excitation induced by irritation of external source. All secondary activity of odontoblasts results only in secondary deposits which are always irregular and erratic in structure; probably the nearest approach to regularity in these is the fact of lamination, which merely shows eras of activity.

The form of secondary calcification most frequently met with is that known as secondary dentine, discoverable to some extent in canals and pulp chambers of most persons beyond maturity. Secondary dentine may be pretty evenly distributed over the walls of a pulp chamber, or confined to a particular section of the wall, or appear in the form of tumors or nodulations, more or less pedunculated, firmly attached to the cavity wall. The benefits and evils of secondary dentine are about evenly conferred and well mixed, i. e., neither is universal and absolute.

As already suggested, development of secondary dentine is due to specific irritation of external relation, consequently the greater formations occur on those parts of the wall of the pulp cavity nearest to or toward the source of irritation.

Activity of odontoblasts in secondary calcification may be manifested over a considerable portion of a pulp at once, provided the irritation were general, a condition most likely due to prevalence of extreme temperatures, or probable alternation of them. Whilst the odontoblasts are most actively engaged thus, the individual probably feels no discomfort, and the progress of deposition may be so slow as to require years before producing noticeable effect; on the contrary, very extensive secondary deposits may occur in a year or less time.

Secondary calcification is usually more rapid than regular dentinification. Secondary dentine often occupies nearly all the pulp chamber, causing the reduction of the pulp until it merely exists within the canal in the root.

In rare instances nodules of calcific matter are found in canals; this rarity is probably due to a lowering of vitality in the pulp and thus restricting activity of odontoblasts. There is another reason why odontoblasts within the root canals do not often resume spasmodic activity; they are not subject to external influences to the same extent as those within the crown of the tooth; naturally a root is protected and may be said to have no external relations other than physiological.

A pulp canal is subject to two constrictions, which may be called apexal and coronal; the first constriction to be considered here is the coronal, located at the point where the pulp enters the canal from the chamber; the coronal constriction doubtless is the secondary one, for reasons which may appear farther on. The coronal or secondary constriction is related to practice in important degree; every thorough operator has experienced difficulty in penetrating some canals, because of a spicula or plate of secondary dentine that projects itself over or across the entrance to the canal, sometimes quite closing it, or appearing to; the removal of this obstruction often reveals a liberal canal beyond.

But the coronal constriction is not due to external irritation; it comes in response to natural demand to meet an emergency. Whenever the dentine of the crown has attained maximum thickness for mechanical purposes of a tooth, strength and wear and tear, the activity of the odontoblasts must be reduced and finally stopped, and this only can be done by reducing or lowering the vitality of the pulp itself, enfeebling it but not destroying it. An external irritation may cause enough congestion of the pulp to stimulate renewed activity of the odontoblasts and support them imperfectly for a time, but this spasmodic effort receives only spasmodic support; the supply of pabulum (building material) fluctuates with the wavering inflammation. Inflammation is high living, and, if extreme in a pulp, nutrition fails.

The coronal constriction may be developed so rapidly and extensively as to literally strangle the coronal portion of the pulp, and it dies.

There are other secondary deposits to be observed before reaching the apex of the root. Pulp nodules are oval or rounded bodies frequently found in the substance of pulps, having no connection whatever with the wall of the cavity or any masses of secondary dentine. Doubtless the same character of irritation that stimulates development of secondary dentine also influences formation of independent nodules, but in the latter the odontoblasts have no part, unless possibly one becoming separated sinks into the substance of the pulp and becomes a nucleus around which accretion of lime salts occurs from the superabundance drawn to the pulp by mild irritation, and which the coronal constriction prevents being promptly returned to the general circulation. The process of nodulation within pulp tissue is analogous to that by which oysters construct pearls.

While nodules within canals are rarely discovered, the writer has had the good fortune to discover quite a number, in one instance three in a canal (of an inferior bicuspid). Some cut specimens of pulp nodules show beautiful laminations that evidently mark periods of activity and repose in the process of calcific deposition.

Going back again to the pulp chamber, from which we wandered in quest of pearls, let us penetrate the tubuli for farther discoveries of lime deposits. Under stress of certain conditions, the pulp fails to invest against encroachments by deposits within its cavity, but instead the embryoplastic filaments occupying the tubuli affected by an irritant permit or accomplish transmission of lime salts in solution, the filaments operating as osmotic tissue for transmission of fluid to the peripheral loops. The lime salts thus passing through the tubules are laid within the terminal sections of the filaments themselves as close to the irritant as possible, thus affecting a complete solidification of that section of dentine; this character of deposit is classed as Obstructive Calcification, and is in nearly every essential different from all other calcific deposits or formations in teeth. This obstructive action may occur solely in a group of tubuli that are directly affected by thermal changes from a metallic filling; this may be easily demonstrated by making a section of dentine along the tubes from a filling or even an unfilled cavity of long standing, the opacity of the affected tubes will be easily observed even by the unaided eye in most cases.

Obstructive calcification also prevails to a good degree in many cases, this being particularly so in senile abrasion, or any very slow process by which dentine is destroyed over living pulps Thus we are enabled to account for the low sensibility often observed in the teeth of the aged. In all these obstructive measures nature acts solely on the defensive against outside influences; the pulp nodules alone being erratic calcification. Probably the order in which these secondary deposits might be anticipated in the same tooth would be, *first*, obstructive calcification; *second*, secondary dentine, and, *third*, pulp nodules.

There is no pulp dynamics capable of nourishing dentine solidified by obstructive calcification.

Penetrating the canal once more, we discover another interesting differentiation in secondary calcification known as canal casts; from their appearance these differ materially from every other secondary formation, although of the same constituency as those named; the casts are formed within canals in apparently the same manner as the nodules, but are long rod-like masses, and conforming closely to the walls of the canals has given the name canal casts. Some canal casts are quite solid, while others appear to be formed of fine crystals, like asbestos; occasionally one appears to be attached to the dentine, but it is seldom more than a close mechanical adaptation. As in case of the pulp nodules, so in the canal casts the odontoblasts have no interest.

It is supposed that the fibrous or spongy casts are so because of a rapid atrophy of the pulp and lowering or expiring vitality in that organ, brought about by cemental constriction of the apexal end of the canal—the primary constriction.

It is well—or ought to be—well known that the apex of a root tooth is always completed by cementum, if completed at all, and in this cemental tissue we have a continuance of the process of calcification that began with the appearance of stellate bodies in the enamel organ. The study of this subject is not finished until the process ends, which does not occur until the pulp is dead; even then it does not lose its significance, because the want of additional layers to lengthen the root in order to compensate for abrasion and to maintain articulation, makes the loss a doubtful advantage.

The histories of the dental pulp and progressive dental calcification are written in the same book and must be read together. Reading them thus brings us to the following conclusions:

First. Time enough being given, the pulp will inevitably destroy itself, or be obliterated by influences of progressive calcification.

Second. From the stage of completion of a tooth, the pulp labors toward self-destruction, by secondary deposits.

Third. After practical completion (articulation) of a tooth the tenure does not depend upon continued vitality of the pulp.

Fourth. While unnecessary destruction of pulps should be discouraged it is better to attain certainty of good results with pulpless canals than wrestle with disappointing after-effects of attempted conservation of live pulps.

Fifth. A pulp that can not live without protection should be protected; pulps that can not survive without protection were better destroyed.

DISCUSSION.

PROF. TAFT: I believe that wherever there is living organic tissue it must be nourished. There is a portion of organic tissue in dental canals and there is a nutritive process going on. We frequently find that the teeth increase in density which may be due to a deposition of calcific material between the canals. Whether there is progressive increase in density as age progresses, is a question. Nutritive currents contain calcareous matter which becomes deposited on the walls of the canals, thus closing them up. Dentine under certain conditions and circumstances does exhibit an increasing density. The deposition of calcific matter upon the walls of the pulp-chamber is a provision of nature to resist both wear upon the occluding surfaces of the crowns and the encroachment of disease.

DR. A. W. HARLAN: Dr. Cravens' paper discusses itself. One or two conclusions are, I think, a little startling. He makes the statement that a pulp which can live without protection should be saved and one that can not live without protection should be protected. In cases of extreme mechanical abrasion the pulp retreats, for the odontoblast is a progressive builder. If extensive gold cappings are put on the pulp frequently dies. Though I have not looked up the literature of the subject, I agree with the general trend of the paper. In these cases of extensive abrasions mentioned it is probably best in many instances to obliterate the pulp in order to conserve the integrity and color of the teeth. Subject passed.

